# Wide and increasing suitability for *Aedes albopictus* in Europe is congruent across distribution models

**DOI:** 10.1038/s41598-021-89096-5

**Published:** 2021-05-10

**Authors:** Sandra Oliveira, Jorge Rocha, Carla A. Sousa, César Capinha

**Affiliations:** 1 Centre for Geographical Studies, Institute of Geography and Spatial Planning, Universidade de Lisboa, Lisbon, Portugal; 2 Global Health and Tropical Medicine, GHTM, Instituto de Higiene e Medicina Tropical, IHMT, Universidade Nova de Lisboa, Lisbon, Portugal

**Keywords:** Risk factors, Infectious diseases, Ecology, Ecological epidemiology

## Abstract

The Asian tiger mosquito (*Aedes albopictus*), a vector of dengue, Zika and other diseases, was introduced in Europe in the 1970s, where it is still widening its range. Spurred by public health concerns, several studies have delivered predictions of the current and future distribution of the species for this region, often with differing results. We provide the first joint analysis of these predictions, to identify consensus hotspots of high and low suitability, as well as areas with high uncertainty. The analysis focused on current and future climate conditions and was carried out for the whole of Europe and for 65 major urban areas. High consensus on current suitability was found for the northwest of the Iberian Peninsula, southern France, Italy and the coastline between the western Balkans and Greece. Most models also agree on a substantial future expansion of suitable areas into northern and eastern Europe. About 83% of urban areas are expected to become suitable in the future, in contrast with ~ 49% nowadays. Our findings show that previous research is congruent in identifying wide suitable areas for *Aedes albopictus* across Europe and in the need to effectively account for climate change in managing and preventing its future spread.

## Introduction

Vector-borne diseases are a worldwide burden that is projected to rise due to climate change. *Aedes albopictus* is a competent vector of several diseases of global concern, such as dengue fever and the Zika and Chikungunya viruses^1^. Originally confined to Southeast Asia, this species has spread notoriously over the last few decades, driven by the globalization of travel and trade, and is now present in all populated continents^2,3^. The invasion process is still ongoing in many newly colonized regions, with vast areas expected to become colonized in the near future^4,5^. Climate change is expected to further aggravate these settings, particularly at temperate latitudes, where regions that are currently too cold for sustaining the species could soon become suitable^6–8^.

In Europe, *Ae. albopictus* first arrived in 1979 in Albania and in 1990 in Italy^9^. Currently, the species is established in more than 20 countries^10^ and it has been responsible for outbreaks of dengue and Chikungunya in Croatia, France, and Italy^9^. Public health concerns have spurred research on the suitability of the European continent to the establishment of the species. Under current conditions, predictions reveal a seemingly consistent pattern of suitability along the southern coastal regions of European Mediterranean countries^11–13^, while some studies also predict suitable conditions in central Europe^14^ and further north in the southern British Isles^15,16^. In future conditions, predictions indicate an increase in suitability in the Balkans^17^, eastern Europe, and southern areas of Scandinavia^7, 18,19^, although these results are highly variable among studies.

Most published predictions of habitat suitability for *Ae. albopictus* are derived from statistical models relating the species’ known distribution to spatial predictors. These models share a common theoretical background^20^, but their implementation differs across studies. The differences include, for example, the use of distinct sources of subsets of the species distribution data, such as VBORNET (European Network for Arthropod Vector Surveillance for Human Public Health)^19^, VectorMap^8^, or the global compendium of the *Aedes* species^12,21^ or the use of different modeling algorithms, including MaxEnt^6,22^, other machine-learning techniques^11^ and fuzzy logic^7^. The procedures for model calibration can also vary substantially, and include the use of background points^8^ or pseudo-absence points^11^ to contrast with species observations, and the use of distinct sets of predictors. For example, while all models use climatic predictors, a few also consider human-related factors, such as urbanization levels or socioeconomic gradients^5,13,23^. Importantly, observed differences in model implementation reflect distinct, yet plausible, hypotheses about the appropriate mathematical construct of the species habitat suitability^20^. Accordingly, previously published predictions of habitat suitability for *Ae. Albopictus* in Europe can reasonably be assumed as equally valid estimates of the true potential distribution of the species^24^. Given the perceived variability in results from published studies, a pressing need is, therefore, to identify areas where inter-model consensus is high, which could more confidently inform public health policies, as well as to determine areas of inter-model disagreement and where further modeling efforts are desirable.

Here, we assess and map levels of consensus among published predictions of habitat suitability for *Ae. albopictus* in Europe. We classify the level of agreement and uncertainty according to the number of matching results, aggregate them in three major categories and apply a color scheme equivalent to a traffic light system to facilitate interpretation. We apply this procedure to two distinct timeframes, namely, the present-day timeframe, representing current climatic conditions, and to a future period referring to predictions based on climate change projections centered in 2050. With this simplified classification, we quantify the prevalence of nine trajectories of environmental suitability. Finally, considering that the most densely populated areas are more exposed to vector-borne diseases^25^, we assessed the suitability for the species and the expected variation in the future, for a set of major urban areas in Europe.

## Materials and methods

### Input data models

We performed a literature search in the Web of Science (WOS), Google Scholar, and through contacts with experts, to identify studies with statistical-based estimates of the environmental suitability (also known as potential distribution) of *Aedes albopictus* covering Europe. The specific criteria used to identify suitable modeling studies are available in the supplementary information. The identified studies include model predictions for either present-day conditions, future conditions based on climate change projections, or both. Present-day conditions refer to the potential distribution of *Ae. albopictus* for a period of at least 10 years between the 1950 and 2014 time range. Future conditions refer to the spatial distribution of *Ae. albopictus* projected for the period centered in 2050 based on climate change scenarios. In total, we obtained 7 independent predictions of suitability for current conditions and 5 for scenarios of future conditions (Table 1).

**Table 1:** Main characteristics of the input models and corresponding references.

References models	Geog. coverage	Spatial resolution	Present-day period	Future period	Scenario	Modelling technique
Caminade et al. (2012^19^)	Europe	0.25° ~ 25 km	1960–2009	2030–2050	SRES A1B	GIS-based (overwintering and seasonal activity); Multi-criteria decision analysis
Campbell et al. (2015)^8^	Global	0.16666° ~ 18 km	1950–2000	2041–2060	SRES B1	MaxEnt
Ding et al. (2018)^11^	Global	0.05° ~ 5 km	1970–2000			Support vector machine (SVM); Gradient boosting machine (GBM); random Forest (RF)
Kraemer et al. (2015^12^, 2019^5^)	Global	0.04166° ~ 5 km	1960–2014	2050	RCP 6.0	Boosted regression trees (BRT)
Proestos et al. (2015)^7^	Global	0.46875° ~ 50 km	2000–2009	2045–2054	SRES A2	Fuzzy-logic
Rogers (2015)^52^	Global	0.5° ~ 55 km	1961–1990	2080 (estimated for 2050 by linear interpolation)	SRES B1	K-means clustering; Nonlinear discriminant analysis
Santos and Meneses, (2017)^13^	Global	30 arc-sec ~ 1 km	1950–2000			MaxEnt

*RCPs* representative concentration pathways, *SRES* Special Report on Emissions Scenarios, *GIS* Geographic Information Systems. The scenario corresponds to the data layer used in our analysis. The original works may include results from other scenarios that were not analyzed, and these are detailed in the corresponding works cited.

All the procedures carried out to pre-process, harmonize and classify the data were developed in R software^26^, and spatial analysis was done in combination with ArcGIS 10.6.1, from ESRI.

### Classifying suitable and unsuitable conditions

The predictions obtained from published studies were in continuous scales (either 0 to 1 or 0 to 100), representing the probability of conditions being suitable to the species. Because the meaning of probability values can differ strongly between distinct models^27^, we classified the areas in each prediction into suitable or unsuitable to the species using the presence threshold (also known as fixed omission) method^28,29^. This has been a widely adopted criterion to classify probability values of species distribution models into suitable or unsuitable conditions (e.g.,^30,31^) and consists in using as the threshold the probability value below which a fixed proportion of observed species occurrences takes place (e.g., 5%, 10%). The reason for not using the probability value at which no occurrences are omitted is that some of these records may not represent established populations (e.g. sporadic individuals or population sink areas)^29^.

To perform the classification, we overlapped the known occurrences of *Ae. albopictus* in Europe given by the Global Compendium of *Aedes aegypti* and *Ae. albopictus* occurrence,^21^ to each prediction obtained for current conditions. We then identified probability values below which 5% of species occurrences take place, assuming that in the areas where this threshold is lower the conditions are unsuitable to the species. Projections of suitability under future conditions used the same thresholds identified for current conditions for the matching model (Supplementary Information, Table S1). To account for the sensitivity of results to the threshold value adopted, we performed the analysis also using the 10th percentile^28^. In the main text, we present the results obtained using the 5% threshold, whereas those based on the 10% threshold are provided as supplementary information.

### Harmonizing the spatial resolution of input models

The predictions had different spatial resolutions, ranging from about 1 km to 50 km (Table 1), which required a spatial harmonization step. For this purpose, we created a new grid of square cells with a resolution of 25 km to conciliate the different cell sizes to an intermediate dimension, whose conversion procedure is described in the supplementary materials. The geographic extent of the new grid, with 8679 cells, matches the smaller extent common across all models and covers most of continental Europe, except the northern parts of Scandinavia, eastern Europe, and Russia.

### Defining categories of consensus suitability and uncertainty

Starting with the seven models for present-day conditions, we calculated levels of inter-model consensus in the classification of suitability. This calculation resulted from the sum of models after the suitable conditions were coded as 1 and unsuitable conditions as 0. The cells with a higher number of matching models (i.e., having sum values close to 0 or to 7) were identified as the areas where the predicted class is consensual. On the contrary, cells whose summed values approached the middle point of the sum range reflect a strong inter-model disagreement in the predicted class. As such, we assumed that the level of uncertainty regarding the suitability (or unsuitability) for the mosquito decreases when the number of matching models is higher, whereas uncertainty is greatest when about half of the models agree. With these criteria, we defined three categories that relate suitability classes and the level of uncertainty: (1) “unsuitable with low uncertainty”, where most models (at least 5 out of 7) agree on unsuitability; (2) “suitable with low uncertainty”, where most models (at least 5 out of 7) agree on suitability; (3) “high uncertainty”, where only 3 or 4 of the models agree for either class.

For scenarios of future conditions, we adopted a similar approach. In this case, the limits of the three categories were adjusted to the combination of 5 spatial predictions. The category of higher uncertainty was defined when only 2 or 3 of the models predict the same class (corresponding to about half of the models), whereas the high agreement categories had to include the match of at least 4 of the 5 models, for either suitable or unsuitable.

### Identifying hotspots of suitability for *Ae. albopictus* in Europe and potential future trajectories

We identified hotspots of consensus based on the number of matching inter-model predictions. This was first done separately for current conditions and for future conditions. A second step consisted of determining the variation of suitability expected between the two timeframes. These trends were identified through the changes in the main categories combining the suitability and uncertainty levels, where 1 represents unsuitable with low uncertainty; 2 represents high uncertainty; and 3 represents suitable with low uncertainty. We identified nine possible trajectories of suitability change. Three of these reflect the maintenance or increase in unsuitability, 3 represent changes towards uncertainty, and 3 others indicate that suitability in the future is maintained or instead increased from either unsuitable or uncertain categories in present-day conditions.

For the mapping and visual representation of the major categories and the future trajectories, we used a scheme based on a traffic light system, which is a color scheme classification that has equivalent meanings in different fields and is easily interpreted by the public^32^. In this scheme, green corresponds to the most favorable situation from the human viewpoint, i.e., unsuitable for the mosquito with low uncertainty, yellow corresponds to an intermediate situation (high uncertainty regarding either suitability and unsuitability) and red indicates the most negative situation, with suitability for the mosquito being consensual across models. The variations found amongst categories between the present-day and future conditions are represented by transitional colors between the gradients of the 3 main ones (Supplementary Information, Table S2).

### Assessing future trajectories of *Ae. albopictus* suitability in urban areas

We analyzed the suitability for *Ae. albopictus* in 65 large functional urban areas (FUA), corresponding to cities and a surrounding commuting zone with generally more than 250,000 inhabitants^33^. The selection procedure is described in detail in the supplementary information. The patterns verified in the present-day conditions and the trends for the future are illustrated separately for each urban area, using the traffic light scheme, according to a baseline scenario, by assigning to each FUA the category with wider spatial coverage. In some cases, a part of the FUA was covered by a category whose variation would result in a more unfavorable trajectory than that given by the baseline scenario. In these cases, and when the second largest category occupied at least a third of the urban area, we adopted a cautious approach and recalculated the variations considering a worst-case scenario.

## Results

### Identifying hotspots of suitability for *Ae. albopictus* in Europe under current and future conditions

**Figure 1:**
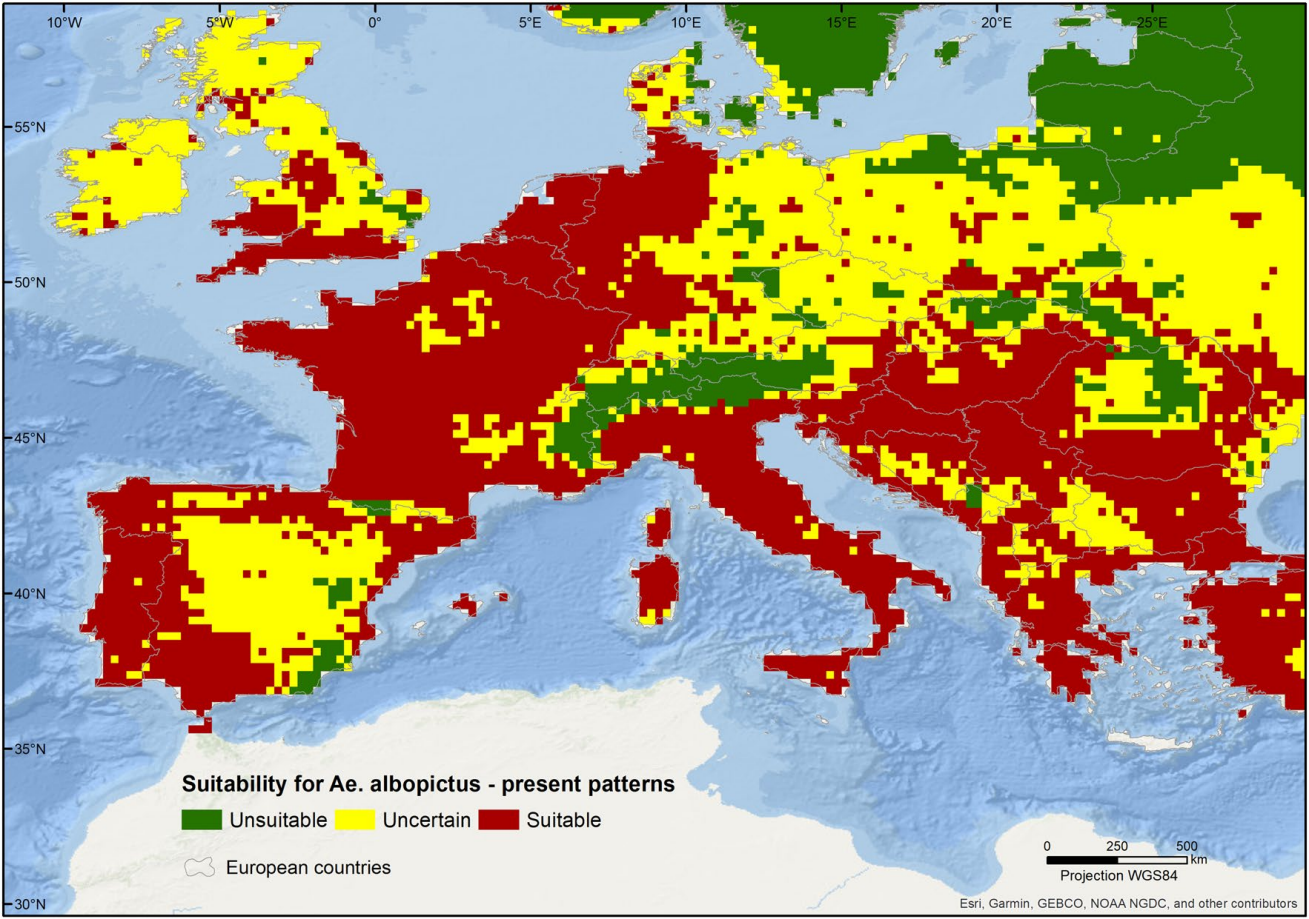
Patterns of consensus among published predictions of current habitat suitability for *Aedes albopictus* in Europe. Suitable and unsuitable areas result from the agreement of 5 or more predictions. The map was created using ArcGIS v. 10.6.1 (https://www.arcgis.com/).

Measurements of inter-model consensus indicate that, presently, suitable conditions occur mainly in the southern and western areas of the continent, extending to central Europe up to the southern edge of Great Britain (Fig. 1).

**Figure 2:**
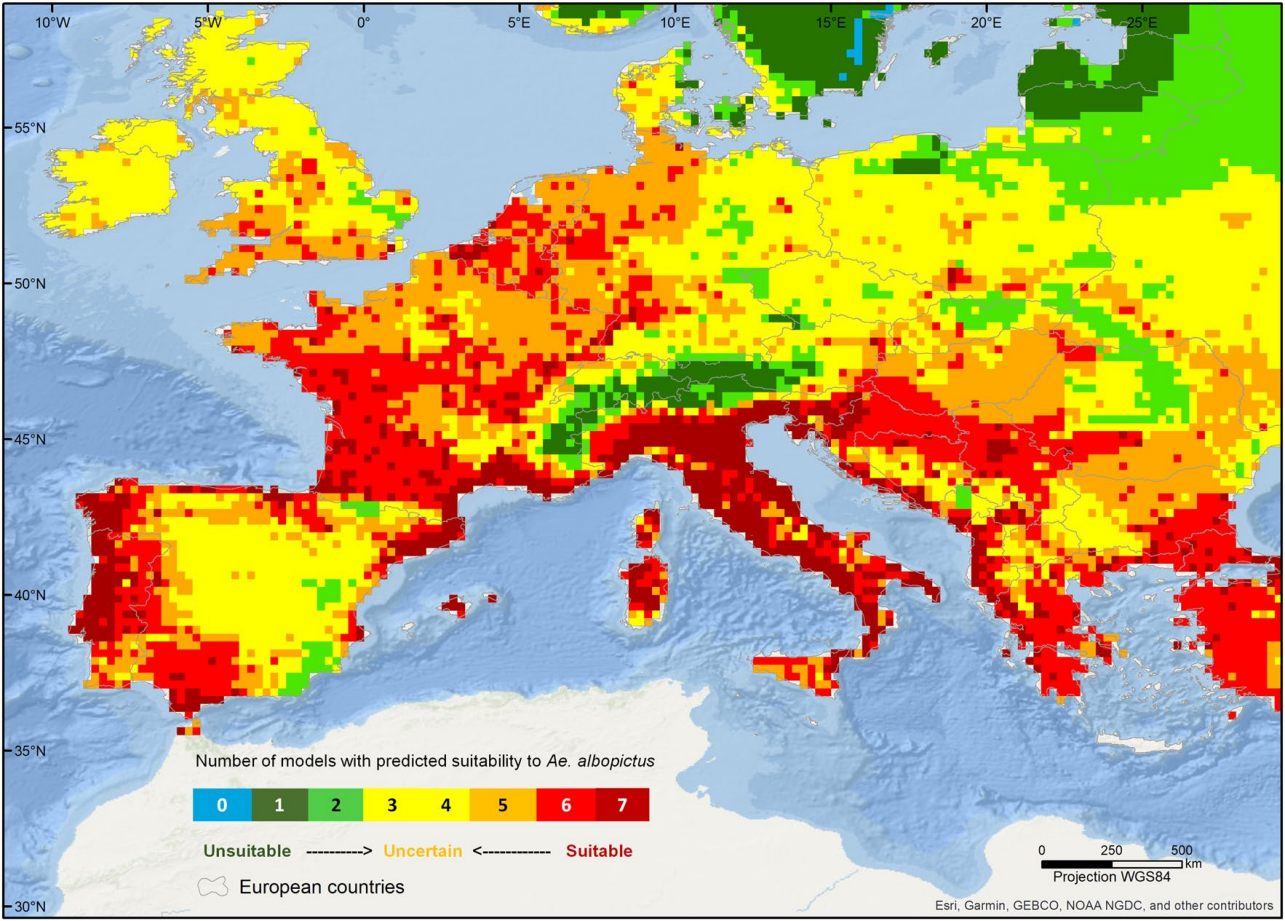
Levels of agreement among published predictions of habitat suitability for *Aedes albopictus* under present-day conditions. Agreement corresponds to the sum of binary suitability maps, with suitable areas coded as one and unsuitable areas as zero. The map was created using ArcGIS v. 10.6.1 (https://www.arcgis.com/).

The highest concordance regarding suitable areas, with a match of all predictions, is observed for the northwest of the Iberian Peninsula, southern France, most of mainland Italy and in parts of the Mediterranean coastline, between the western Balkans and Greece (Fig. 2). For the south of Great Britain and most of the north of central Europe, suitability is supported by 5 out of 7 models (Fig. 2). The Scandinavian and Baltic regions, as well as the mountainous areas of the Alps, the Pyrenees, and the Carpathians, are predicted as unsuitable with high certainty. In Scandinavia and in the Alps, at least 6 of the models agree on the unsuitability to the species. Areas of high uncertainty, which reflect a high disagreement between the predictions, occur mainly in eastern Europe, northern Britain, Ireland, and central Spain (Fig. 2). Results based on the 10^th^ percentile threshold show wider areas of unsuitability, particularly in central Europe (Supplementary Information, Figs. S2 and S3).

**Figure 3:**
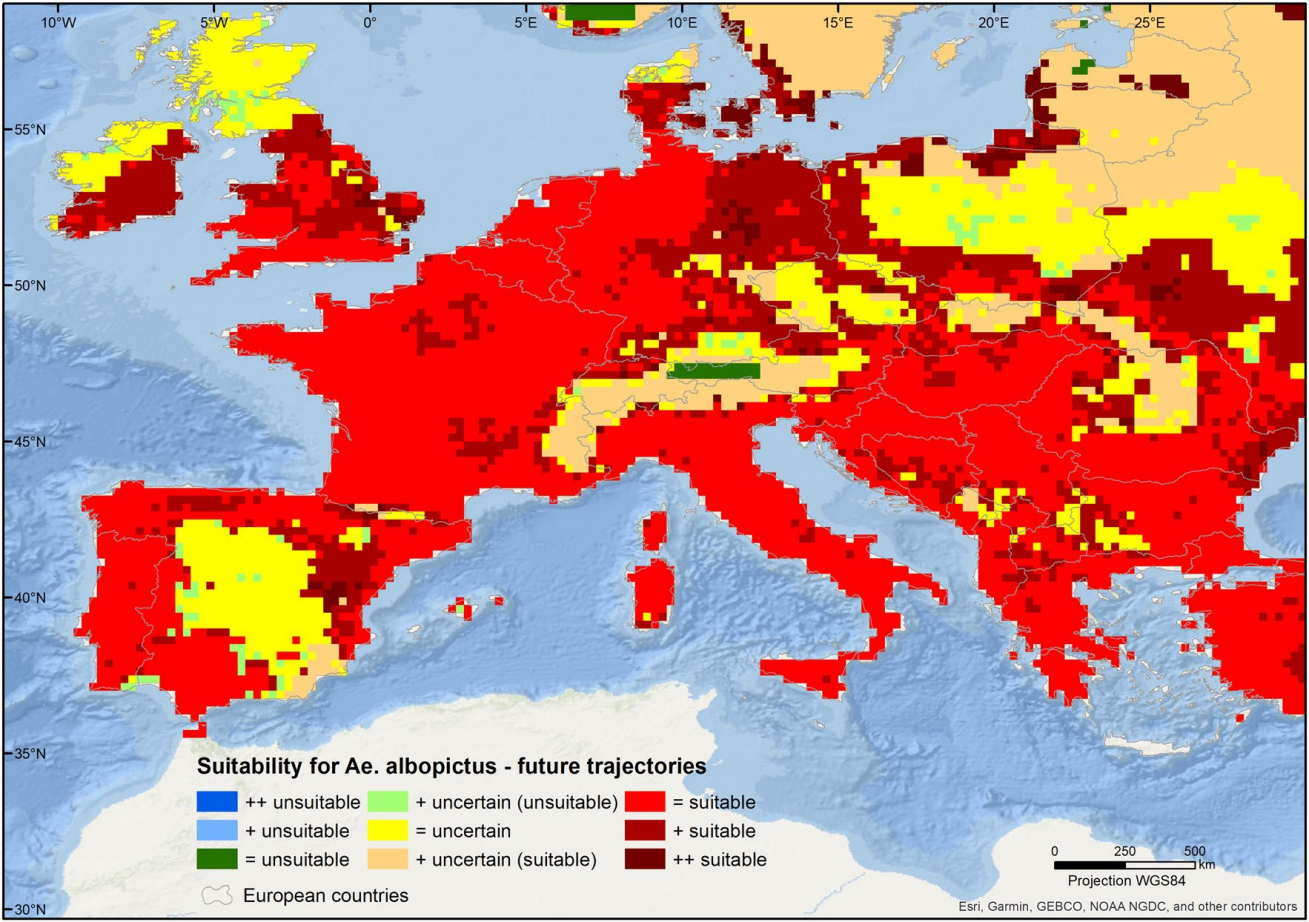
Future trajectories of suitability for *Aedes albopictus* in Europe. Each trajectory represents a different combination of predicted status (suitable, uncertain, unsuitable) in the two timeframes (present and future). The map was created using ArcGIS v. 10.6.1 (https://www.arcgis.com/).

The patterns of consensus-based suitability for the present-day timeline markedly change under future climatic conditions. These changes follow different trajectories within the European continent, depending on the estimated variation between the two timeframes amongst the 3 categories (unsuitable with low uncertainty, high uncertainty, and suitable with low uncertainty, Table S2). Patterns of consensus obtained using the 5th percentile indicate that, in the future, suitable regions will encompass 21% more area, adding to the 47% of the continent that is predicted to remain suitable (Fig. 3). About 2.5% of the newly suitable areas will take place in areas currently predicted as unsuitable, specifically in the southwest coast of Sweden and in the north coast of the Baltic countries. The change from other classes to unsuitable does not occur and only 0.54% of the study area will remain unsuitable. Overall, unsuitable areas decrease to only a few spots, located in the higher Alps, Scandinavia, and in the central Iberian Peninsula (Fig. 3). Conversely, suitable areas, with high certainty, expand further north, reaching central Great Britain, southern areas of Ireland and Denmark, and southwestern areas of Sweden. The extent of regions with high uncertainty increases by 16%, mostly in areas classified as unsuitable in the present day. This means that roughly half of the models support that currently unsuitable regions, such as Scandinavia and Baltic countries, can become suitable in the future. Results based on the less conservative threshold, show similar spatial patterns, albeit punctuated by wider areas of uncertain suitability (Supplementary Information, Figs. S4 and S5).

### Trends of suitability for *Ae. albopictus* in urban areas

**Figure 4:**
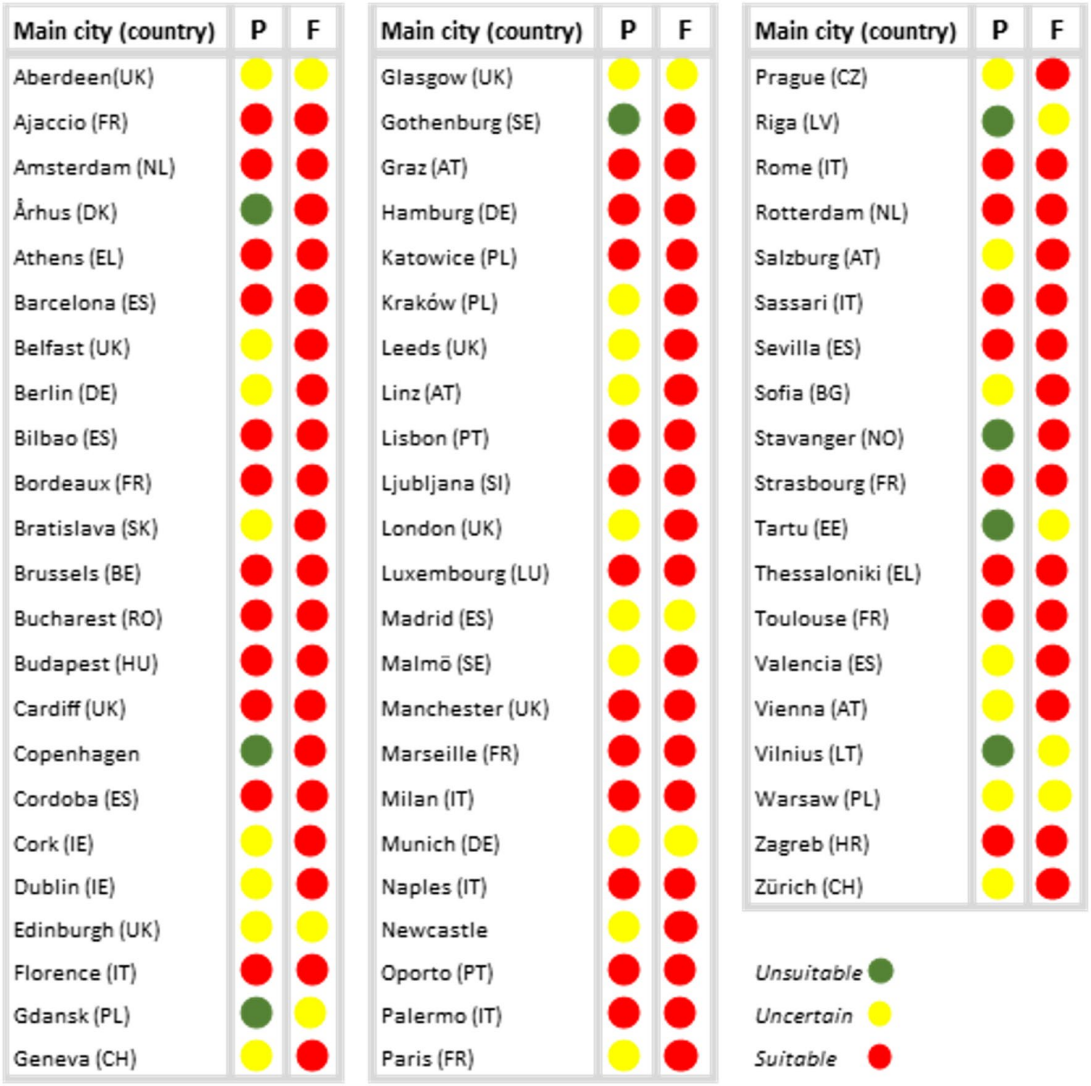
Present and future suitability for *Aedes albopictus* in functional urban areas of major European cities. P represents present-day conditions; F represents future conditions. Colors follow a traffic-light scheme, with green corresponding to the most favorable situation from the human point-of-view (unsuitable with low uncertainty), red as the most negative situation (suitable with low uncertainty), and yellow as the intermediate situation (high uncertainty).

Presently, 49% of 65 major European urban areas predicted to be suitable to the species, 39% of them are of uncertain suitability and 12% are predicted as unsuitable. The numbers change noticeably for future conditions, where 83% of urban areas are predicted as suitable by the majority of individual predictions, while none are predicted as unsuitable (Fig. 4). The remaining 17% of urban areas are predicted as uncertain in the future, with at least half the models indicating possible suitable conditions. Cities located in northern Europe, such as Arhus, Copenhagen, Gothenburg, and Stavanger, are expected to undergo the most severe changes, going from unsuitable with high certainty in the present day to suitable with high certainty in the future (Fig. 4). Cities of central Europe, Great Britain, and Ireland, such as Belfast, Berlin, Dublin, Geneva, London, Prague, and Vienna, are expected to become suitable for the establishment of the species as time progresses, whereas suitability in cities such as Edinburgh, Madrid, or Warsaw remains uncertain. The results obtained for the 10% probability threshold show a similar marked tendency for an increase in suitability for the mosquito, although of a lower magnitude (Supplementary Information, Fig. S6).

## Discussion

*Aedes albopictus* is a competent vector for diseases of epidemic potential and the widening of its distribution in Europe raises public health concerns. We evaluated the level of consensus amongst published predictions regarding the environmental suitability for this mosquito species in Europe, considering both present-day and future climatic conditions. Our results show that most predictions agree on the suitability of the southern and western regions of Europe for the species. The high consensus on suitability for these regions is not unexpected, given that their climatic conditions largely match the ones occupied by the species in its current range^5–8,11,13, 18,19,22^. Suitability is also predicted for central Europe, the Balkans, and the south of the Great Britain, albeit with lower inter-model support. In the last few years, the species has been recorded in some of these regions^9,15,16^, inclusively leading to outbreaks of dengue and Chikungunya in France and of dengue in Croatia ^9^. On the contrary, strong disagreement between predictions was found for northern Great Britain, Ireland, and eastern Europe. These locations largely coincide with the transition between the warmer to mild climates found in the south and in coastal areas, and the cold climates of high latitudes, mountainous areas and Eastern Europe, where harsh winters of the continental climate hinder the survival of the species. The finding that the models strongly disagree for these areas may be explained by differences in the predictors used for each model, specifically those associated to the tolerance limits of the mosquito regarding climatic variables (e.g., temperature limits for mosquito development). The topic itself encompasses some uncertainty, as striking differences on ranges of tolerance to climatic factors have been recorded (e.g., cold) for populations from different regions^34^. Thus, discrepant areas can represent regions where the species may have the capacity to occur temporarily, eventually depending on the periodicity or duration of abnormal weather. Testing this hypothesis and assessing the extent to which the occurring individuals reach densities of epidemiological concern, will require further research.

### Future trajectories of suitability to *Aedes albopictus*

According to our results, in about 30 years, *Ae. albopictus* will find suitable areas in 68% of the European continent, including most of the British Isles, Ireland, and the southern areas of Scandinavian countries. Most models agree with the future expansion of the mosquito to the northern and eastern regions and no decrease in suitability has been found for any region. This estimated change in suitability for the mosquito will increase the risk of vector-borne diseases, even in places where this risk is now completely absent. The likely expansion of the geographic distribution of vectors and the consequent rise in the human incidence of related diseases has also been discussed by prior research^9,35–40^. Moreover, the predicted future range of the expansion of the mosquito has not taken into account the rapid adaptation traits of this species, which has the ability to colonize different ecological niches ^1,3^. Predictive models are based on currently known environmental requirements and consider them to be fixed over relevant timescales, when they in fact can be subject to rapid evolutionary changes^41^.

These scenarios call for the implementation of efficient systems of surveillance and control, supported by international cooperation^4,42^. Considering the adaptability of the species, no single approach is likely to work everywhere and different alternatives will need to be tested^43,44^. Public health policies will need to adjust to potential threats brought about by the increase in vector availability, which can trigger outbreaks of Chikungunya, dengue, Zika, or other viruses. Additionally, health systems in currently unaffected countries will have to prepare and integrate preventive and mitigating measures to control the spread of vector-borne diseases. Indeed, the establishment of the mosquito will also depend on other factors, such as the availability of breeding sites, the density of human settlements, and the availability of feeding hosts ^1,9^. Some authors have also found encouraging signs, as the policies adopted to limiting a temperature increase of 2 °C are expected to restrain the extent of the conditions conducive to the *Aedes* species expansion and, consequently, dengue cases^45^. Also, under the effect of increased dryness and heat, southern European countries may become less suitable for the mosquito^19,20^, which could explain the uncertainty found for central Spain. Furthermore, although some areas are suitable for the species (i.e., areas that form permanent self-sustaining populations), different regions can sustain populations presenting markedly distinct patterns of seasonality^46^, which display a different epidemiological potential for transmitting diseases.

### The suitability of urban areas in Europe

The implications of the establishment of *Ae. albopictus* will depend on its degree of contact with human populations^13,44,47,48^. The Asian tiger mosquito is adapting to urban environments^1,44^ and our findings show that the suitability of the mosquito will increase in most cities. A few areas considered unsuitable with high certainty today will become suitable, which is the case for Copenhagen and Gothenburg. In central Europe, cities like Berlin, Geneva, and Prague will undergo a consensual change towards higher suitability, similar to what is expected for Dublin and London. Urban densely populated areas can support the establishment of the mosquito via the heat island effect, with the rise in urban temperature amplifying climate change effects, and in addition by the supply of mosquito breeding sites in man-made water containers and through irrigation^25,49,50^. Moreover, the higher availability of potential hosts in urban areas and the dynamics of urban movements increase the risk of disease spread^49,51^. Our findings suggest that the potential exposure of people to vector-borne diseases will be much higher in the future, a troubling pattern that is particularly critical in northern European cities.

The use of research outputs resulting from different modelling procedures required the transformation and simplification of the original data, and the uncertainties of each original model could be absorbed by our combined maps. To ensure the compatibility of all the available predictions, the resolution of some of the maps was reduced and the details specific to each model were lost. However, the increase of the accessible information and the divergences found amongst studies, make the transfer of scientific outputs into tangible and consensual policies a challenging task. Our research allowed to identify hotspots of high and low suitability for *Ae. albopictus* in Europe, as well as the areas that currently show a high inter-model mismatch. These findings could be used to highlight more pressing areas of research, to clarify the uncertainty levels in specific regions, or to improve our understanding of the factors driving the expansion to the areas where the mosquito is currently absent, but where it is estimated to appear in the future, particularly in urban areas.

## Supplementary Information

Supplementary Information 1.

## Data Availability

The analyzed data are available in an anonymized form from: https://doi.org/10.5281/zenodo.4721245. Original predictions of the species distribution were obtained through direct contacts with the corresponding authors of relevant publications and so are not publicly available. These data are however available from the authors upon reasonable request and with permission of Cyril Caminade, David J. Rogers, Fangyu Ding, Moritz U. G. Kraemer, Townsend Peterson and Yiannis Proestos.
