# Supplementary material for: Wide and increasing suitability for Aedes albopictus in Europe is congruent across distribution models
Source: Sci Rep. 2021 May 10;11:9916. doi: 10.1038/s41598-021-89096-5 (PMC8110805; doi:10.1038/s41598-021-89096-5)
Supplement: Supplementary file 1 — Supplementary Information 1. [file 41598_2021_89096_MOESM1_ESM.pdf]

SUPPLEMENTARY MATERIAL

Wide and increasing suitability for Aedes albopictus in Europe is congruent across distribution models

Sandra Oliveira[1], Jorge Rocha[1], Carla A. Sousa[2], and César Capinha[1*]

[1] Centre for Geographical Studies, Institute of Geography and Spatial Planning, Universidade de Lisboa, Lisbon, Portugal.

[2] Global Health and Tropical Medicine, GHTM, Instituto de Higiene e Medicina Tropical, IHMT, Universidade Nova de Lisboa, Universidade Nova de Lisboa, Lisbon, Portugal.

*Corresponding author (cesarcapinha@campus.ul.pt)

Materials and Methods

Input data models

The following criteria were used to identify suitable modeling studies, while performing the literature search:

i) Spatial predictions were presented separately for Ae. albopictus, even if other species were also investigated;
ii) The predictions exclusively concerned the distribution of the species, rather than the diseases it can transmit;
iii) The minimum geographical coverage was continental Europe;
iv) The temporal coverage of the studies could be present-day or future time periods;
v) For projections under future conditions, these would have to be based on the IPCC standard scenarios (families A and B or representative concentration pathways (RCPs), [1], assuming that the different scenarios provide climate change projections and model parameters with equivalent representativeness.

The future models presented estimations for the period around 2050, except in one case whose predictions referred to 2080. In this case, the values for 2050 were estimated based on linear interpolation, considering the expected evolution since 1990 (the present-day timeframe of the corresponding model).

After identifying studies fitting our criteria, we obtained the original spatial predictions either directly from the study (e.g., if provided as a supplementary information) or by contacting the corresponding authors.

Identifying thresholds of presence/absence

Table S1. Cutoff values obtained for each model based on two different percentiles, applied for both present and future conditions.

References/models	5%	10%
Caminade et al., 2012 [2]	0.3918694	0.6355554
Campbell et al., 2015 [3]	0.001107	0.001938
Ding et al., 2018 [4]	0.4592	0.6576
Kraemer et al., 2015 [5]; 2019 [6]	0.1398	0.1912
Proestos et al., 2015 [7]	0.1345	0.270
Rogers et al., 2015 [8]	0.01	0.01
Santos & Meneses, 2017 [9]	0.0178	0.0418

Harmonizing the spatial resolution of input models

After creating the new grid with a resolution of 25 km, we assigned to each cell the values of every prediction, converting them from its original resolution (between ~1 and 50 km). From the models with a coarser resolution, the new grid cells were given the values of the overlapping cells. From the models with a finer resolution, the upscaled grid cells received the value of the class that occupied the majority of its area. For the models with an equivalent resolution to the new grid, the values were retrieved from the coinciding cells.

Identifying hotspots of *Ae. albopictus* suitability in Europe and potential future trajectories

The color scheme classification is based on based on a traffic light system; green corresponds to the most favorable situation from the human viewpoint, i.e., unsuitable for the mosquito with low uncertainty, yellow corresponds to an intermediate situation (high uncertainty regarding either suitability and unsuitability) and red indicates the most negative situation, with suitability for the mosquito being consensual across models. The variations found amongst categories between the present-day and future conditions are represented by transitional colors between the gradients of the 3 main ones. Three of the categories, represented in blue and green, reflect the maintenance or increase in unsuitability; 3 other categories represent changes towards uncertainty and are colored in yellow and orange, and 3 others indicate that suitability in the future is maintained or instead increased from either unsuitable or uncertain categories in present-day conditions, represented in red colors.

Table S2. Classification and color scheme defined to represent the major categories and future trajectories of suitability for *Ae. albopictus*

	**Timeframe**		***Trajectory***	
Major categories	***Present***	***Future***	***Code***	***Description***
Unsuitable, low uncertainty	1	1	=	Equally unsuitable
		2	+	Higher uncertainty (towards suitable)
		3	++	Much more suitable
High uncertainty	2	1	+	More unsuitable
		2	=	Equal uncertainty
		3	+	More suitable
Suitable, low uncertainty	3	1	++	Much more unsuitable
		2	+	Higher uncertainty (towards unsuitable)
		3	=	Equally suitable

Assessing future trajectories of Ae. albopictus presence in urban areas

The spatial boundaries of the urban areas were obtained from the 2018 Urban Audit (Fig. S1). They correspond to functional urban areas (FUAs), which consist of densely inhabited cities and a less populated commuting zone, whose labor market is highly integrated in the main city [10,11]. We selected FUAs that were classified as metropolitan (between 250,000 and 1.5 million inhabitants) or large metropolitan areas (above 1.5 million inhabitants. An exception was made for Corsica, Sardinia, and Estonia, for which the FUAs available are classified as medium-sized (between 100,000 and 250,000 people). The option to delimit cities based on FUAs is justified by the high risk of disease spread caused by commuting movements [12–14].

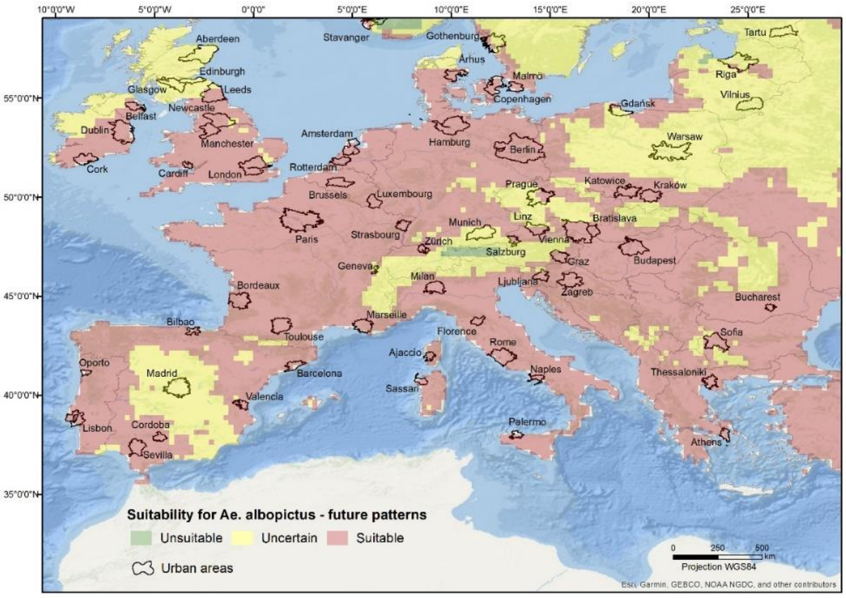

Figure S1. Location and boundaries of the 65 Functional Urban Areas analyzed. The background information shows the future patterns of suitability, classified in the 3 major categories. The map was created using ArcGIS v. 10.6.1 (https://www.arcgis.com/).

References

1. Nakicenovic, N. *et al. Special report on emissions scenarios (SRES), a special report of Working Group III of the intergovernmental panel on climate change.* (2000).

2. Caminade, C. *et al.* Suitability of European climate for the Asian tiger mosquito Aedes albopictus: recent trends and future scenarios. *J. R. Soc. Interface* **9**, 2708–2717 (2012).

3. Campbell, L. P. *et al.* Climate change influences on global distributions of dengue and chikungunya virus vectors. *Philos. Trans. R. Soc. B Biol. Sci.* **370**, 20140135 (2015).

4. Ding, F., Fu, J., Jiang, D., Hao, M. & Lin, G. Mapping the spatial distribution of Aedes aegypti and Aedes albopictus. *Acta Trop.* **178**, 155–162 (2018).

5. Kraemer, M. U. *et al.* The global distribution of the arbovirus vectors Aedes aegypti and Ae. Albopictus. *Elife* **4**, 1–18 (2015).

6. Kraemer, M. U. *et al.* Past and future spread of the arbovirus vectors Aedes aegypti and Aedes albopictus. *Nat. Microbiol.* **4**, 854–863 (2019).

7. Proestos, Y. *et al.* Present and future projections of habitat suitability of the Asian tiger mosquito, a vector of viral pathogens, from global climate simulation. *Philos. Trans. R. Soc. B Biol. Sci.* **370**, 20130554 (2015).

8. Rogers, D. J. Dengue: Recent past and future threats. *Philos. Trans. R. Soc. B Biol. Sci.* **370**, 1–18 (2015).

9. Santos, J. & Meneses, B. M. An integrated approach for the assessment of the Aedes aegypti and Aedes albopictus global spatial distribution, and determination of the zones susceptible to the development of Zika virus. *Acta Trop.* **168**, 80–90 (2017).

10. OECD. *Redefining Urban: A New Way to Measure Metropolitan Areas*. (2012).

11. EUROSTAT (ESTAT) GISCO, E. C. Urban Audit 2018. [Retrieved from https://ec.europa.eu/eurostat/web/gisco/geodata/reference-data/administrative-units-statistical-units/urban-audit#ua18]. (2018).

12. Sanna, M. & Hsieh, Y. H. Ascertaining the impact of public rapid transit system on spread of dengue in urban settings. *Sci. Total Environ.* **598**, 1151–1159 (2017).

13. Wen, T. H., Lin, M. H. & Fang, C. T. Population Movement and Vector-Borne Disease Transmission: Differentiating Spatial-Temporal Diffusion Patterns of Commuting and Noncommuting Dengue Cases. *Ann. Assoc. Am. Geogr.* **102**, 1026–1037 (2012).

14. LaDeau, S. L., Allan, B. F., Leisnham, P. T. & Levy, M. Z. The ecological foundations of transmission potential and vector-borne disease in urban landscapes. *Funct. Ecol.* **29**, 889–901 (2015).

Results – supplementary figures

Identifying hotspots of suitability for Ae. albopictus in Europe under current and future conditions

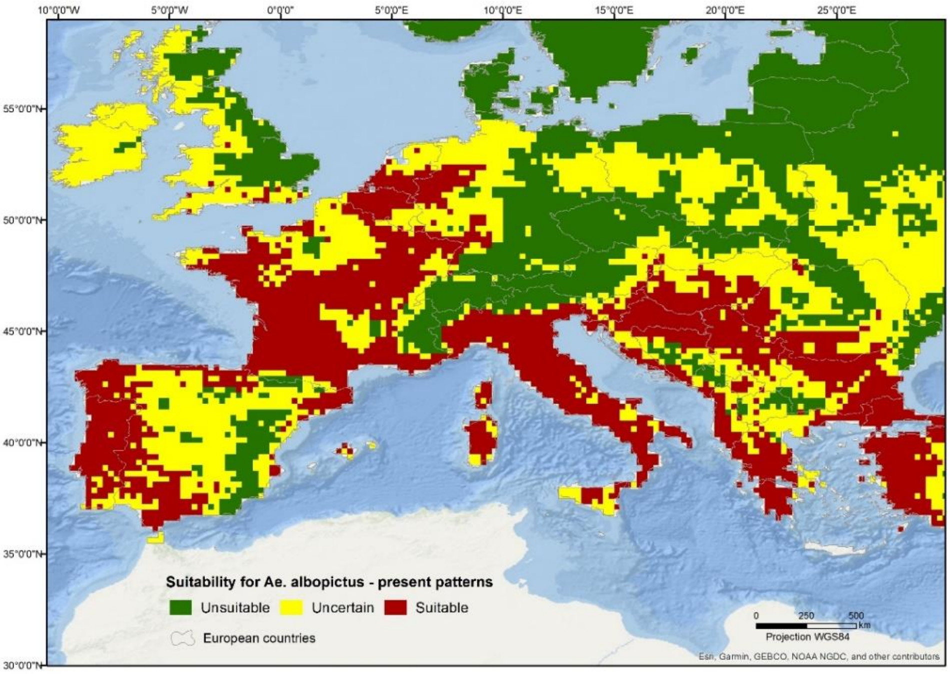

Figure S2. Inter-model agreement of predictions of habitat suitability for *Aedes albopictus* under present-day conditions, using the 10% threshold. The map was created using ArcGIS v. 10.6.1 (https://www.arcgis.com/).

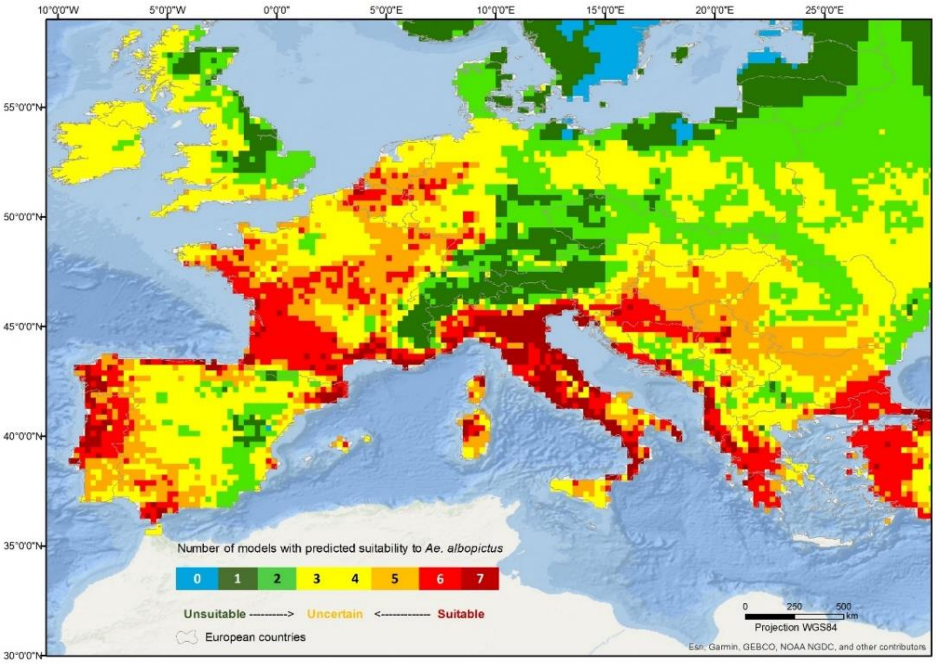

Figure S3. Number of models with predicted suitability to *Aedes albopictus* (coded as 1), using the 10% threshold. The map was created using ArcGIS v. 10.6.1 (https://www.arcgis.com/).

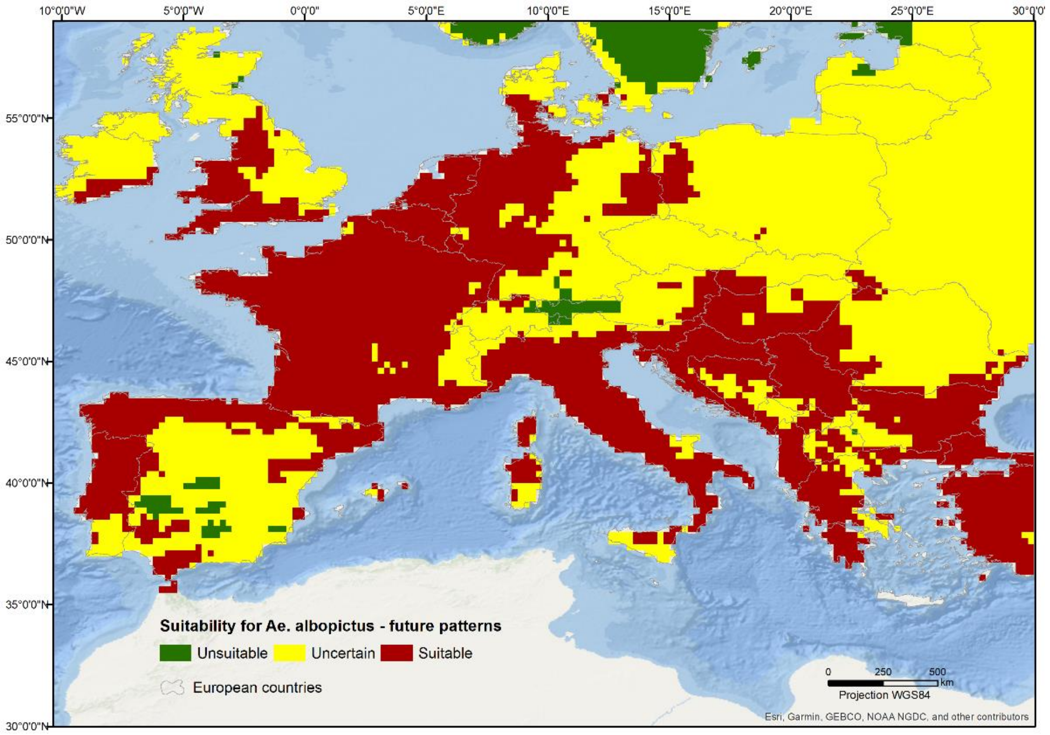

Figure S4. Inter-model agreement of predictions of habitat suitability for Aedes albopictus under future climatic conditions, using the 10% threshold. The map was created using ArcGIS v. 10.6.1 (https://www.arcgis.com/).

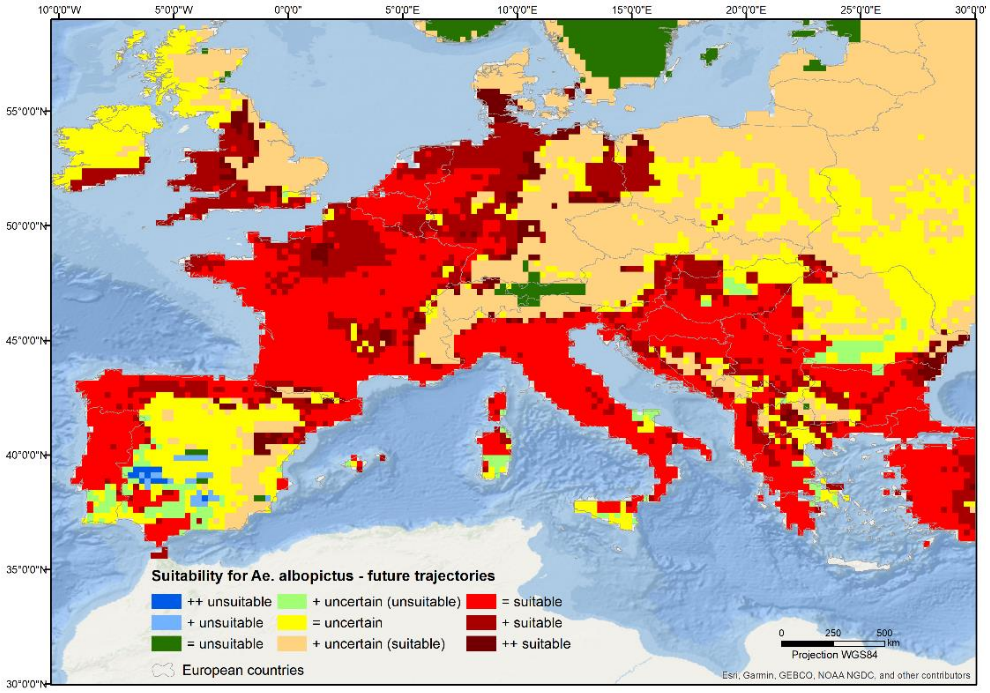

Figure S5. Future trajectories of suitability to Ae. albopictus in Europe, using the 10% threshold. The map was created using ArcGIS v. 10.6.1 (https://www.arcgis.com/).

Trends of suitability for *Ae. albopictus* in urban areas

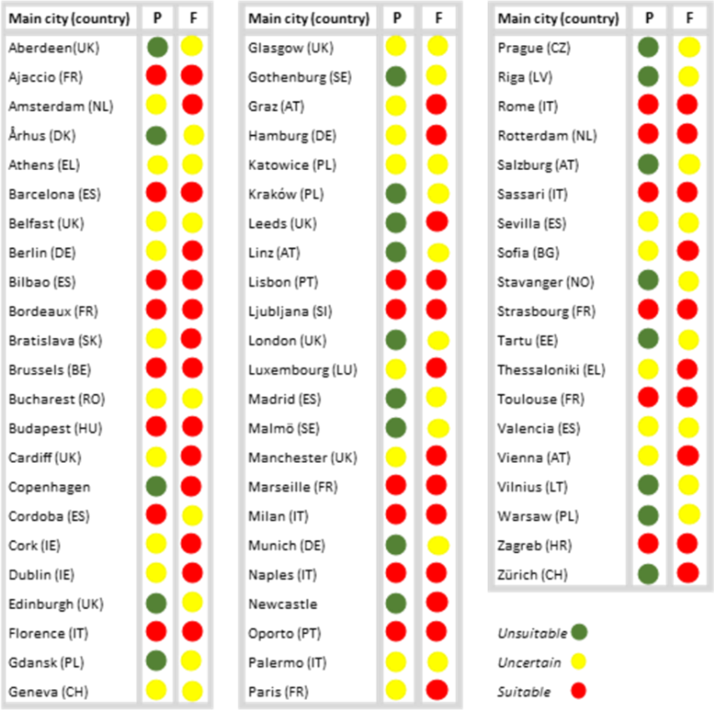

Figure S6. Present and future suitability for *Ae. albopictus* in major European cities (Functional Urban Areas), considering the 10% threshold. P represents present-day conditions; F represents future conditions. Colors follow a traffic-light scheme, with green corresponding to the most favorable situation from the human point-of-view (unsuitable with low uncertainty), red as the most negative situation (suitable with low uncertainty), and yellow as the intermediate situation (high uncertainty).
